# Supplementary material for: Trends in Influenza Vaccination Rates among a Medicaid Population from 2016 to 2021
Source: Vaccines (Basel). 2023 Nov 11;11(11):1712. doi: 10.3390/vaccines11111712 (PMC10675465; doi:10.3390/vaccines11111712)
Supplement: Supplementary file 1 [file vaccines-11-01712-s001.zip › vaccines-2658131-supplementary.pdf]

SUPPLEMENTAL MATERIAL

Figure S1: Sub-season vaccination rate for individuals who were vaccinated in the given influenza season.

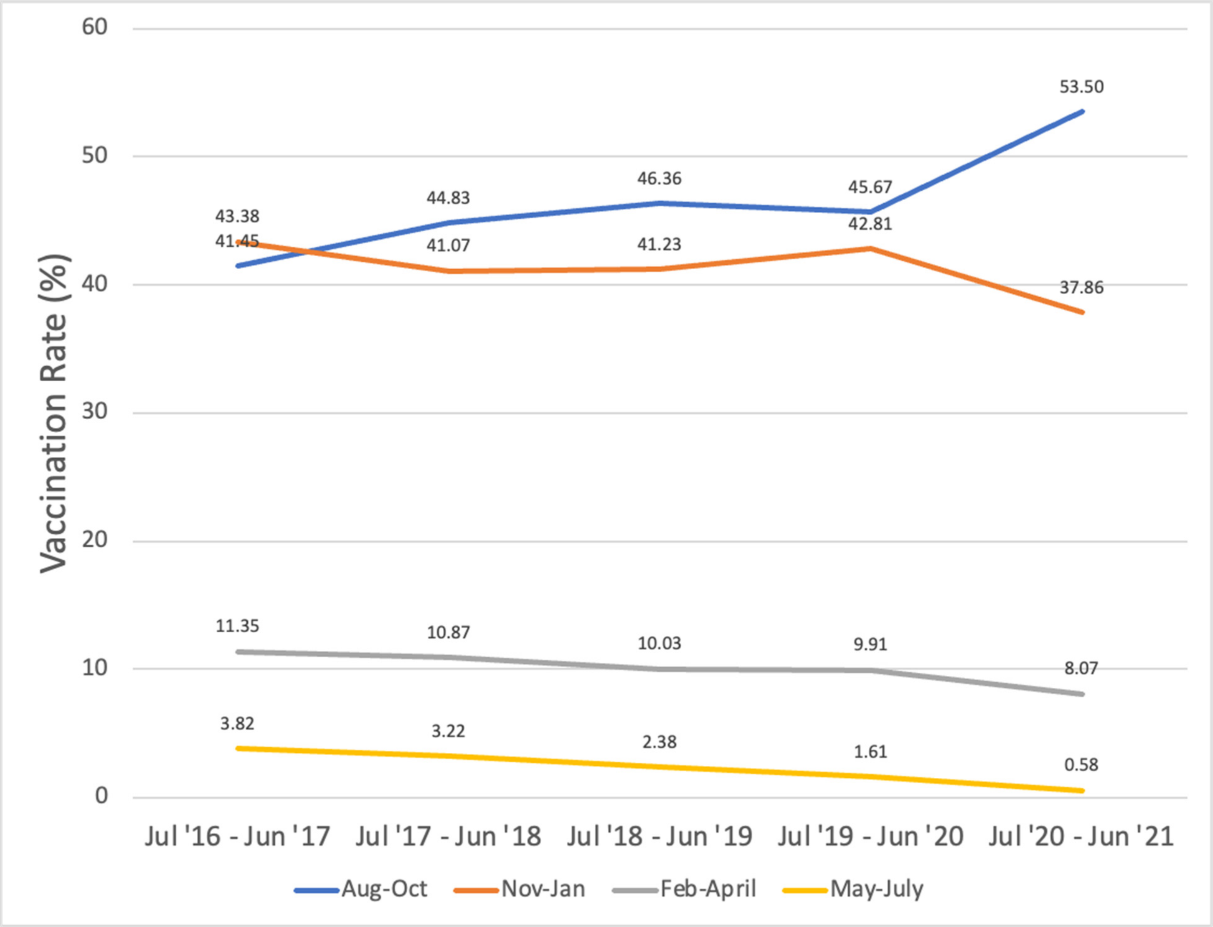

Table S1: List of ICD-9 and ICD-10 codes to identify enrollees who had diabetes in the study population.

Diabetes 1	E10*	ICD-10
Diabetes 2	E11*	ICD-10
Unspecified Diabetes	E08*, E09*, E13*	ICD-10
Diabetes 1	250*(1|3) (the code is given in RegEx)	ICD-9
Diabetes 2	250*(0|2) (the code is given in RegEx)	ICD-9
Unspecified Diabetes	O24* and '250	ICD-9

Table S2: List of ICD-9 and ICD-10 codes to identify enrollees who were pregnant in the study population.

Pregnancy	Z34*, Z36*, O09*, Z3201	ICD-10
Delivery	P95*, Z38*, Z37*, O60*, O82*, O80*, O75*, Z3A*, O4201	ICD-10
Pregnancy	V7242, V283*, V22*, V23*	ICD-9
Delivery	V27*, V30*, V31*, V32*, V34*, V36*, V37*, V39*	ICD-9

Table S3: Rural-Urban Continuum Codes (RUCC) and Descriptions.

RUCC	Description	Category
1	Counties in metro areas of 1 million population or more	Metropolitan
2	Counties in metro areas of 250,000 to 1 million population	Metropolitan
3	Counties in metro areas of fewer than 250,000 population	Metropolitan
4	Urban population of 20,000 or more, adjacent to a metro area	Non-Metropolitan
5	Urban population of 20,000 or more, not adjacent to a metro area	Non-Metropolitan
6	Urban population of 2,500 to 19,999, adjacent to a metro area	Non-Metropolitan
7	Urban population of 2,500 to 19,999, not adjacent to a metro area	Non-Metropolitan
8	Completely rural or less than 2,500 urban population, adjacent to a metro area	Non-Metropolitan
9	Completely rural or less than 2,500 urban population, not adjacent to a metro area	Non-Metropolitan

Table S4: List of CPT codes used to determine flu vaccine uptake.

Codes	Vaccine Codes & Descriptors
90470	H1N1 immunization administration (intramuscular, intranasal), including counseling when performed
90630	Influenza virus vaccine, quadrivalent (IIV4), split virus, preservative free, for intradermal use
90653	Influenza vaccine, inactivated (IIV), subunit, adjuvanted, for intramuscular use
90654	Influenza virus vaccine, trivalent (IIV3), split virus, preservative-free, for intradermal use
90655	Influenza virus vaccine, trivalent (IIV3), split virus, preservative free, when administered to children 6-35 months of age, 0.25ml dosage for intramuscular use
90656	Influenza virus vaccine, trivalent (IIV3), split virus, preservative free, when administered to individuals 3 years and older, 0.5mL dosage, for intramuscular use
90657	Influenza virus vaccine, trivalent (IIV3), split virus, when administered to children 6-35 months of age, 0.25 mL dosage, for intramuscular use
90658	Influenza virus vaccine, trivalent (IIV3), split virus, when administered to individuals 3 years of age and older, 0.5ml dosage, for intramuscular use
90659	Influenza virus vaccine, whole virus, for intramuscular or jet injection use

90660	Influenza virus vaccine, trivalent, live (LAIV3), for intranasal use
90661	Influenza virus vaccine (ccIIV3), derived from cell cultures, subunit, preservative and antibiotic free, for intramuscular use
90662	Influenza virus vaccine (IIV), split virus, preservative free, enhanced immunogenicity via increased antigen content, for intramuscular use
90663	Influenza virus vaccine, pandemic formulation, H1N1
90664	Influenza virus vaccine, live (LAIV), pandemic formulation, for intranasal use
90666	Influenza virus vaccine (IIV), pandemic formulation, split virus, preservative free, for intramuscular use
90667	Influenza virus vaccine, pandemic formulation, split virus, adjuvant, for intramuscular use
90668	Influenza virus vaccine (IIV), pandemic formulation, split virus, for intramuscular use
90672	Influenza virus vaccine, quadrivalent, live (LAIV4), for intranasal use
90673	Influenza virus vaccine, trivalent (RIV3), derived from recombinant DNA, hemagglutinin (HA) protein only, preservative and antibiotic free, for intramuscular use
90674	New in 2017: Influenza virus vaccine, quadrivalent (ccIIV4), derived from cell cultures, subunit, preservative and antibiotic free, 0.5 mL dosage, for intramuscular use
90682	New in 2017: Influenza virus vaccine, quadrivalent (RIV4), derived from recombinant DNA, hemagglutinin (HA) protein only, preservative and antibiotic free, for intramuscular use
90685	Influenza virus vaccine, quadrivalent (IIV4), split virus, preservative free, when administered to children 6-35 months of age, 0.25ml dosage, for intramuscular use
90686	Influenza virus vaccine, quadrivalent (IIV4), split virus, preservative free, 0.5 mL dosage, for intramuscular use
90687	Influenza virus vaccine, quadrivalent (IIV4), split virus, when administered to children 6-35 months of age, for intramuscular use
90688	Influenza virus vaccine, quadrivalent (IIV4), split virus, when administered to individuals 3 years of age and older, 0.5 ml dosage, for intramuscular use
90689	Influenza virus vaccine quadrivalent (IIV4), inactivated, adjuvanted, preservative free, 0.25mL dosage, for intramuscular use
90694	Influenza virus vaccine, quadrivalent (aIIV4), inactivated, adjuvanted, preservative free, 0.5 mL dosage, for intramuscular use
90724	Influenza virus vaccine
90756	Influenza virus vaccine, quadrivalent (ccIIV4), derived from cell cultures, subunit, antibiotic free, 0.5 mL dosage, for intramuscular use
G0008	Administration of influenza virus vaccine
G8108	Patient documented to have received influenza vaccination during influenza season.

G8482	Influenza immunization administered or previously received
G8639	Influenza immunization was administered or previously received
G9141	Influenza A (H1N1) immunization administration (includes the physician counseling the patient/family)
G9142	Influenza A (H1N1) vaccine, any route of administration
Q2033	Influenza vaccine, recombinant hemagglutinin antigens, for intramuscular use
Q2034	Influenza virus vaccine, split virus, for intramuscular use (agriflu)
Q2035	Influenza virus vaccine, split virus, when administered to individuals 3 years of age and older, for intramuscular use (afluria)
Q2036	Influenza virus vaccine, split virus, when administered to individuals 3 years of age and older, for intramuscular use (flulaval)
Q2037	Influenza virus vaccine, split virus, when administered to individuals 3 years of age and older, for intramuscular use (fluvirin)
Q2038	Influenza virus vaccine, split virus, when administered to individuals 3 years of age and older, for intramuscular use (fluzone)
Q2039	Influenza virus vaccine, not otherwise specified
V0481	Need for prophylactic vaccination and inoculation against influenza.
V066	Need for prophylactic vaccination and inoculation against streptococcus pneumoniae [pneumococcus] and influenza

Table S5: Number of enrollees who had at least one healthcare encounter (N) and vaccination rates (%) stratified by flu seasons.

Category	Jul '16 – Jun '17<br>N(%)	Jul '17 – Jun '18<br>N(%)	Jul '18 – Jun '19<br>N(%)	Jul '19 – Jun '20<br>N(%)	Jul '20 - Jun '21<br>N(%)	P-value**
**Enrollees with 1+ Encounters, N**	3,167,653	4,834,338	4,530,306	4,275,226	4,061,387	-
**Total Enrollees Vaccinated**	349,993 (11.05)	669,645 (13.85)	688,626 (15.20)	699,380 (16.36)	642,827 (15.83)	
**Age group**						
6 months - 4 years	310,191 (23.36)	549,639 (26.01)	555,466 (29.70)	592,139 (30.69)	557,855 (30.93)	<0.0001
5-17 years	1,006,662 (11.62)	1,578,325 (16.10)	1,498,394 (17.13)	1,373,693 (18.62)	1,249,369 (18.41)	<0.0001
18-64 years	1,850,800 (8.68)	2,706,374 (10.07)	2,476,446 (10.78)	2,309,394 (11.34)	2,254,163 (10.66)	<0.0001
**Gender**						
Female	1,719,173 (11.54)	2,667,642 (14.07)	2,523,536 (15.33)	2,398,109 (16.39)	2,288,273 (15.78)	<0.0001
Male	1,448,480 (10.47)	2,166,696 (13.58)	2,006,770 (15.04)	1,877,117 (16.32)	1,773,114 (15.89)	<0.0001
**Metropolitan Area**						
Metropolitan	2,423,215 (11.08)	3,861,983 (13.91)	3,623,989 (15.28)	3,448,526 (16.40)	3,328,512 (15.84)	<0.0001
Non-Metropolitan	744,438 (10.93)	972,355 (13.63)	906,317 (14.89)	826,700 (16.20)	732,875 (15.78)	<0.0001
**Greater than median higher education area**						
Yes	1,416,138 (11.05)	2,193,694 (13.92)	2,039,096 (15.28)	1,918,601 (16.43)	1,843,321 (15.83)	<0.0001
No	1,751,515 (11.05)	2,640,644 (13.79)	2,491,210 (15.14)	2,356,625 (16.30)	2,218,066 (15.83)	<0.0001
**Medicaid Managed Care**						
With Managed Care Program	573,150 (13.32)	1,860,218 (15.55)	1,813,880 (17.01)	1,731,513 (17.98)	1,559,059 (16.92)	<0.0001
Free-for-service	2,594,503	2,974,120	2,716,426	2,543,713	2,502,328	<0.0001

	(10.55)	(12.79)	(13.99)	(15.26)	(15.15)	
**At-risk conditions**						
Diabetes*	555,273 (14.27)	797,018 (17.70)	751,733 (17.08)	651,786 (17.50)	592,472 (15.51)	<0.0001
Diabetes Type 1 (DM 1)	95,807 (17.34)	127,358 (21.00)	120,380 (19.96)	104,747 (19.05)	94,623 (17.01)	<0.0001
Diabetes Type 2 (DM 2)	527,699 (14.42)	756,266 (17.87)	712,947 (17.21)	617,617 (17.61)	560,345 (15.60)	<0.0001
Pregnancy	252,018 (15.39)	405,966 (17.29)	339,479 (16.77)	268,975 (17.21)	203,434 (16.31)	<0.0001

*Enrollees in this category were diagnosed as either: (1) "DM 1 and DM 2", (2) "DM 1 or DM2", or (3) "unspecified diabetes."
** P values were obtained from the chi-square test to show the significance of vaccination rates across influenza seasons.

Table S6: Adjusted Odds Ratio (AOR) and 95% Confidence Intervals for features associated with flu vaccination based on the CCS features

Feature	Overall (Jul'16-Jun'21)	Pre-COVID (Jul'16-Jun'20)	Peri COVID (Jul'20-Jun'21)
**Demographic Characteristics**			
7 months - 4 years (vs. 18-65 years)	3.40 (3.37, 3.42)	3.46 (3.43, 3.48)	2.88 (2.86, 2.91)
5-17 years (vs. 18-65 years)	2.11 (2.10, 2.12)	2.10 (2.09, 2.11)	1.92 (1.91, 1.94)
Male (vs. Female)	0.95 (0.94, 0.95)	0.95 (0.95, 0.96)	0.94 (0.93, 0.94)
Area with higher education (vs. not)	1.00 (1.00, 1.01)	1.01 (1.01, 1.01)	1.00 (0.99, 1.00)
Metropolitan (vs. Non-Metropolitan)	1.02 (1.01, 1.02)	0.99 (0.99, 1.00)	0.99 (0.98, 1.00)
Medicaid managed care (vs. free-for-service)	1.16 (1.16, 1.17)	1.20 (1.19, 1.20)	1.12 (1.11, 1.12)
**Health Conditions**			
Factors Influencing Health Status and Contact with Health Services	2.82 (2.80, 2.83)	2.54 (2.53, 2.56)	2.72 (2.69, 2.75)
Certain Conditions Originating in the Perinatal Period	1.86 (1.85, 1.88)	1.47 (1.46, 1.48)	1.63 (1.61, 1.64)
Diseases of the Eye and Adnexa	1.36 (1.35, 1.36)	1.36 (1.36, 1.37)	1.10 (1.10, 1.11)
Endocrine, Nutritional and Metabolic Diseases	1.28 (1.28, 1.29)	1.28 (1.27, 1.28)	1.19 (1.19, 1.20)
Symptoms, Signs and Abnormal Clinical and Laboratory Findings, Not Elsewhere Classified	1.27 (1.26, 1.28)	1.23 (1.23, 1.24)	1.08 (1.07, 1.09)
Neoplasms	1.27 (1.26, 1.27)	1.27 (1.26, 1.27)	1.24 (1.23, 1.25)
Diseases of the Skin and Subcutaneous Tissue	1.21 (1.21, 1.22)	1.20 (1.19, 1.20)	1.12 (1.12, 1.13)
Diseases of the Digestive System	1.19 (1.19, 1.20)	1.19 (1.18, 1.19)	1.13 (1.12, 1.13)
Diseases of the Respiratory System	1.18 (1.18, 1.19)	1.20 (1.20, 1.21)	1.02 (1.01, 1.03)
Diseases of the Ear and Mastoid Process	1.15 (1.15, 1.16)	1.17 (1.16, 1.17)	1.02 (1.02, 1.03)
Certain Infectious and Parasitic Diseases	1.14 (1.13, 1.14)	1.14 (1.14, 1.15)	1.04 (1.03, 1.04)
Diseases of the Nervous System	1.12 (1.12, 1.13)	1.12 (1.12, 1.13)	1.07 (1.06, 1.07)
Injury, Poisoning and Certain Other Consequences of External Causes	1.12 (1.11, 1.12)	1.13 (1.12, 1.13)	0.96 (0.96, 0.97)
Pregnancy, Childbirth and the Puerperium	1.10 (1.09, 1.10)	1.12 (1.11, 1.13)	0.77 (0.77, 0.78)
Diseases of the Genitourinary System	1.04 (1.04, 1.05)	1.05 (1.05, 1.06)	0.97 (0.96, 0.97)
Diseases of the Musculoskeletal System and Connective Tissue	1.02 (1.02, 1.03)	1.04 (1.03, 1.04)	0.93 (0.92, 0.93)
External Causes of Morbidity	1.02 (1.01, 1.02)	1.00 (1.00, 1.00)	0.90 (0.89, 0.91)

Diseases of the Blood and Blood Forming Organs and Certain Disorders Involving the Immune Mechanism	1.00 (1.00, 1.01)	1.03 (1.02, 1.03)	1.02 (1.01, 1.03)
Mental, Behavioral and Neurodevelopmental Disorders	0.99 (0.98, 0.99)	1.00 (0.99, 1.00)	0.86 (0.85, 0.86)
Diseases of the Circulatory System	0.97 (0.96, 0.97)	0.99 (0.98, 0.99)	0.94 (0.93, 0.95)

Table S7. Adjusted Odds Ratio (AOR) and 95% Confidence Intervals (CI) for all CCSR features used in the logistic regression model with patient number and vaccination prevalence

**CCSR Features**	**AOR**	**95% CI Lower**	**95% CI Upper**	**Patients**	**Prevalence (%)**
Medical examination/evaluation	2.071	2.062	2.08	4011557	49.91
Liveborn	1.785	1.77	1.801	323914	4.03
Antenatal screening	1.342	1.327	1.358	384196	4.78
Encounter for observation and examination for conditions ruled out (excludes infectious disease, neoplasm, mental disorders)	1.287	1.281	1.292	1761838	21.92
Neoplasm-related encounters	1.265	1.257	1.272	971743	12.09
Encounter for mental health conditions	1.255	1.243	1.267	212995	2.65
Malposition, disproportion or other labor complications	1.217	1.201	1.233	195313	2.43
Refractive error	1.215	1.21	1.22	2075303	25.82
Cataract and other lens disorders	1.212	1.2	1.224	253987	3.16
Other specified upper respiratory infections	1.206	1.201	1.211	2568811	31.96
Other specified encounters and counseling	1.184	1.178	1.189	1321378	16.44
Other specified status	1.181	1.176	1.186	2616233	32.55
Abnormal findings without diagnosis	1.175	1.169	1.181	1761838	21.92
Other specified and unspecified skin disorders	1.161	1.154	1.167	946023	11.77
Mental and substance use disorders in remission	1.158	1.146	1.17	231482	2.88
Symptoms of mental and substance use conditions	1.156	1.146	1.167	290960	3.62
Sleep wake disorders	1.152	1.145	1.158	962902	11.98
Disorders of lipid metabolism	1.136	1.129	1.143	969332	12.06
Personal/family history of disease	1.134	1.128	1.14	1358351	16.9
Supervision of high-risk pregnancy	1.131	1.118	1.144	283726	3.53
Obesity	1.128	1.123	1.134	1370407	17.05
Other general signs and symptoms	1.127	1.122	1.132	2106650	26.21
Other specified complications in pregnancy	1.126	1.114	1.139	434029	5.4
Blindness and vision defects	1.125	1.117	1.132	511190	6.36
Acquired foot deformities	1.125	1.113	1.136	221837	2.76
Viral infection	1.125	1.12	1.131	1257077	15.64
Maternal outcome of delivery	1.117	1.104	1.131	442870	5.51
Intestinal infection	1.113	1.104	1.123	310250	3.86
Other specified inflammatory condition of skin	1.111	1.104	1.117	740261	9.21
Cornea and external disease	1.11	1.104	1.116	1001482	12.46
Respiratory signs and symptoms	1.107	1.102	1.112	2672496	33.25
Miscellaneous mental and behavioral disorders/conditions	1.106	1.095	1.116	251576	3.13
Noninfectious gastroenteritis	1.105	1.096	1.113	405094	5.04
Allergic reactions	1.105	1.1	1.11	1723257	21.44
Maternal care related to fetal conditions	1.104	1.091	1.117	271670	3.38
Asthma	1.104	1.098	1.11	978977	12.18
Other specified and unspecified nutritional and metabolic disorders	1.102	1.094	1.11	385000	4.79
Benign neoplasms	1.102	1.095	1.11	593173	7.38
Genitourinary signs and symptoms	1.096	1.09	1.103	996660	12.4

Abdominal pain and other digestive/abdomen signs and symptoms	1.091	1.086	1.097	2417704	30.08
Other specified bone disease and musculoskeletal deformities	1.09	1.08	1.099	280511	3.49
Other specified and unspecified gastrointestinal disorders	1.087	1.081	1.093	907443	11.29
Neurodevelopmental disorders	1.087	1.081	1.092	1256274	15.63
Urinary incontinence	1.086	1.076	1.095	290960	3.62
Other specified and unspecified disorders of the ear	1.084	1.078	1.091	839927	10.45
Hearing loss	1.083	1.073	1.093	254791	3.17
Musculoskeletal pain, not low back pain	1.082	1.077	1.087	2567204	31.94
Uncomplicated pregnancy, delivery or puerperium	1.08	1.069	1.091	683194	8.5
Fracture of the upper limb, subsequent encounter	1.077	1.063	1.091	199332	2.48
Other aftercare encounter	1.075	1.069	1.082	798935	9.94
Other specified and unspecified lower respiratory disease	1.075	1.066	1.084	369728	4.6
Contraceptive and procreative management	1.075	1.068	1.082	856806	10.66
Other specified and unspecified diseases of kidney and ureters	1.074	1.064	1.084	318288	3.96
Esophageal disorders	1.073	1.066	1.08	856002	10.65
External cause codes: fall; initial encounter	1.073	1.066	1.08	583528	7.26
Fungal infections	1.07	1.062	1.077	520835	6.48
Retinal and vitreous conditions	1.069	1.057	1.08	218622	2.72
Other specified male genital disorders	1.066	1.055	1.077	218622	2.72
Diabetes mellitus with complication	1.063	1.055	1.072	589154	7.33
Open wounds of head and neck, initial encounter	1.061	1.052	1.07	315877	3.93
Diseases of mouth; excluding dental	1.06	1.052	1.068	376158	4.68
Oculofacial plastics and orbital conditions	1.058	1.049	1.068	273277	3.4
Nervous system pain and pain syndromes	1.058	1.051	1.065	840731	10.46
Skin/Subcutaneous signs and symptoms	1.056	1.051	1.061	1229750	15.3
Depressive disorders	1.056	1.05	1.062	1416222	17.62
Other specified eye disorders	1.048	1.038	1.059	217014	2.7
Lifestyle/life management factors	1.048	1.04	1.055	545751	6.79
Osteoarthritis	1.047	1.04	1.055	610856	7.6
Tendon and synovial disorders	1.045	1.036	1.053	417150	5.19
Hemorrhoids	1.045	1.034	1.056	217818	2.71
Socioeconomic/psychosocial factors	1.036	1.029	1.043	503956	6.27
Circulatory signs and symptoms	1.034	1.029	1.04	1198403	14.91
Nervous system signs and symptoms	1.034	1.028	1.04	922714	11.48
Other specified and unspecified liver disease	1.03	1.021	1.039	385803	4.8
Headache; including migraine	1.03	1.025	1.035	1321378	16.44
Otitis media	1.03	1.025	1.035	1167860	14.53
External cause codes: place of occurrence of the external cause	1.026	1.015	1.036	254791	3.17
Open wounds to limbs, initial encounter	1.026	1.019	1.034	471806	5.87
Other specified connective tissue disease	1.026	1.02	1.033	876096	10.9
Other specified and unspecified upper respiratory disease	1.026	1.019	1.034	455730	5.67
External cause codes: other specified, classifiable and NEC; initial encounter	1.024	1.015	1.033	289352	3.6
External cause codes: activity codes	1.024	1.014	1.034	275689	3.43
Nonmalignant breast conditions	1.022	1.014	1.031	329540	4.1

Syncope	1.022	1.013	1.031	319895	3.98
Fluid and electrolyte disorders	1.021	1.013	1.028	710522	8.84
Contact dermatitis	1.021	1.013	1.03	331148	4.12
Gastritis and duodenitis	1.021	1.012	1.03	363298	4.52
Hypertension with complications and secondary hypertension	1.019	1.009	1.03	299801	3.73
Other unspecified injury	1.015	1.01	1.021	1243414	15.47
Pleurisy, pleural effusion and pulmonary collapse	1.014	1.004	1.024	315877	3.93
Diabetes mellitus without complication	1.014	1.007	1.022	712129	8.86
Other specified joint disorders	1.013	1.005	1.02	519227	6.46
Bacterial infections	1.012	1.006	1.017	985407	12.26
Hematuria	1.01	1	1.02	257202	3.2
Nerve and nerve root disorders	1.009	1	1.018	308643	3.84
Cardiac dysrhythmias	1.007	0.998	1.016	359279	4.47
Abdominal hernia	1.007	0.998	1.016	319895	3.98
Pneumonia (except that caused by tuberculosis)	1.003	0.994	1.011	403486	5.02
Encounter for administrative purposes	1.003	0.996	1.01	555396	6.91
Acute and unspecified renal failure	1	0.989	1.011	247557	3.08
Nutritional anemia	0.996	0.987	1.004	333559	4.15
Nausea and vomiting	0.996	0.991	1.002	1396931	17.38
Hepatitis	0.996	0.985	1.007	223444	2.78
Superficial injury; contusion, initial encounter	0.995	0.99	1.001	1044082	12.99
Chronic obstructive pulmonary disease and bronchiectasis	0.995	0.987	1.004	455730	5.67
Fever	0.994	0.989	0.999	1135710	14.13
Skin and subcutaneous tissue infections	0.994	0.989	1	971743	12.09
Anxiety and fear-related disorders	0.994	0.989	1	1549646	19.28
Other specified and unspecified endocrine disorders	0.992	0.983	1.002	269259	3.35
Respiratory failure; insufficiency; arrest	0.988	0.978	0.998	319895	3.98
Sinusitis	0.988	0.983	0.994	914676	11.38
External cause codes: unspecified mechanism	0.987	0.977	0.998	236304	2.94
Diseases of middle ear and mastoid (except otitis media)	0.986	0.976	0.996	222641	2.77
General sensation/perception signs and symptoms	0.985	0.978	0.992	590762	7.35
Implant, device or graft related encounter	0.985	0.979	0.991	843142	10.49
Biliary tract disease	0.983	0.974	0.993	282922	3.52
Nutritional deficiencies	0.983	0.977	0.99	656670	8.17
External cause codes: struck by; against; initial encounter	0.981	0.972	0.99	288549	3.59
Acquired absence of limb or organ	0.976	0.966	0.987	204958	2.55
Other specified nervous system disorders	0.975	0.965	0.985	263632	3.28
Gastrointestinal hemorrhage	0.974	0.965	0.984	292568	3.64
Acute and chronic tonsillitis	0.974	0.966	0.981	353653	4.4
Sprains and strains, initial encounter	0.968	0.962	0.974	1016754	12.65
Essential hypertension	0.967	0.961	0.972	1215282	15.12
Suicidal ideation/attempt/intentional self-harm	0.966	0.956	0.976	257202	3.2
Other specified and unspecified mood disorders	0.966	0.956	0.976	229071	2.85
Nonspecific chest pain	0.965	0.959	0.97	1179917	14.68
Diseases of white blood cells	0.965	0.957	0.974	327933	4.08
Fracture of the lower limb (except hip), initial encounter	0.964	0.954	0.973	245950	3.06
Spondylopathies/spondyloarthropathy (including infective)	0.963	0.957	0.969	1333435	16.59

Trauma- and stressor-related disorders	0.963	0.958	0.969	1080251	13.44
Muscle disorders	0.962	0.954	0.97	452515	5.63
Low back pain	0.961	0.955	0.967	1034436	12.87
Septicemia	0.96	0.949	0.971	244342	3.04
Influenza	0.956	0.95	0.963	540125	6.72
Fracture of the upper limb, initial encounter	0.953	0.943	0.963	351242	4.37
Disorders of teeth and gingiva	0.95	0.945	0.956	662296	8.24
External cause codes: motor vehicle traffic (MVT); initial encounter	0.949	0.939	0.96	212995	2.65
Aplastic anemia	0.945	0.937	0.952	473413	5.89
Thyroid disorders	0.943	0.936	0.949	606837	7.55
Disruptive, impulse-control and conduct disorders	0.94	0.932	0.949	328737	4.09
Dysphagia	0.934	0.925	0.944	242735	3.02
Other specified substance-related disorders	0.931	0.921	0.942	274885	3.42
Neoplasms of unspecified nature or uncertain behavior	0.929	0.919	0.939	208173	2.59
Malaise and fatigue	0.925	0.92	0.931	1026399	12.77
Stimulant-related disorders	0.922	0.912	0.931	355261	4.42
Alcohol-related disorders	0.92	0.913	0.928	521639	6.49
Acute bronchitis	0.915	0.91	0.921	793309	9.87
Other specified female genital disorders	0.914	0.908	0.921	762766	9.49
Urinary tract infections	0.913	0.908	0.919	980585	12.2
Calculus of urinary tract	0.912	0.902	0.923	208977	2.6
Coronary atherosclerosis and other heart disease	0.909	0.9	0.918	309446	3.85
Benign ovarian cyst	0.907	0.897	0.917	209780	2.61
Chronic kidney disease	0.904	0.895	0.914	292568	3.64
Cannabis-related disorders	0.901	0.892	0.91	315877	3.93
Biomechanical lesions	0.9	0.89	0.91	212192	2.64
Heart failure	0.893	0.883	0.903	253183	3.15
Menstrual disorders	0.89	0.884	0.896	667923	8.31
Epilepsy; convulsions	0.888	0.88	0.895	410720	5.11
Tobacco-related disorders	0.873	0.867	0.879	836712	10.41
Bipolar and related disorders	0.871	0.864	0.878	503956	6.27
Schizophrenia spectrum and other psychotic disorders	0.838	0.83	0.845	415543	5.17
Opioid-related disorders	0.833	0.826	0.84	498330	6.2
Inflammatory diseases of female pelvic organs	0.83	0.824	0.836	606033	7.54
Gestational weeks	0.772	0.764	0.781	727401	9.05
No immunization or underimmunization	0.617	0.611	0.624	192901	2.4
